# Completeness and changes in data reporting pharmacological interventions to treat COVID-19

**DOI:** 10.1038/s41598-025-06308-y

**Published:** 2025-07-02

**Authors:** Mia Strikić, Shelly Melissa Pranić

**Affiliations:** 1 Department of Mental Health, Teaching Institute for Public Health Split, Vukovarska 46, 21000 Split, Croatia; 2 Department of Public Health, University of Split School of Medicine, Šoltanska 2A, 21000 Split, Croatia; 3 Cochrane Croatia, Šoltanska 2A, 21000 Split, Croatia

**Keywords:** COVID-19, ClinicalTrials.gov, Randomized controlled trials, SARS-CoV-2, Drug discovery, Health care, Medical research

## Abstract

Reporting transparency is essential because studies that test pharmacological interventions for COVID-19 should be based on reliably reported data across dissemination sources. We conducted a cross-sectional study of reporting of WHO Trial Registration Data Set (TRDS) items in ClinicalTrials.gov RCTs from January 1, 2020, to May 31, 2021. Completeness and changes among WHO TRDS items were investigated, whereby two authors evaluated RCTs independently to reach κ ≥ 0.80. The frequency of incomplete or uninformative information and the frequency of changes that altered the meaning of WHO TRDS items were assessed. There were changes during the conduct of the trial to 19 of 24 WHO TRDS items for 122 RCTs and 68 corresponding publications in peer-reviewed journals. Among the items, there were greater missing data-sharing statements in publications (52/68 [76%]) than at the initial (23/122 [19%]) or last (17/122 [14%]) registration. The reliability of extractions was high (kappa range: 0.80–1.00), where the lowest (kappa = 0.80, 95% CI 0.59–1.00) was for intervention description changes between the latest registered data and data in publications. Our findings emphasize the need for more reliable reporting of data between COVID-19 dissemination sources.

## Introduction

Coronavirus disease 2019 (COVID-19), caused by Severe Acute Respiratory Syndrome Coronavirus 2 (SARS-CoV-2)^1^ became a global health problem since the first case was reported in December 2019, in Wuhan, China^2,3^. COVID-19 was declared a pandemic by the World Health Organization (WHO) on March 11, 2020^4,5^. During the pandemic, the European Medicines Agency (EMA) gave conditional marketing authorization for some COVID-19 treatments as well, based on EU standards for efficacy, quality, and safety data^6^. Additionally, the US Food and Drug Administration (FDA) granted Emergency Use Authorization (EUA) for unapproved products to treat, prevent, or diagnose COVID-19 due to the medical threat the disease imposed^7^. The prescription of remdesivir increased^8^ when the FDA approved remdesivir as the first drug for COVID-19 treatment, in October 2020^9^. Subsequently, the FDA and WHO also approved baricitinib, nirmatrelvir/ritonavir, remdesivir, and tocilizumab for certain child and adult populations to prevent progression of mild or moderate COVID-19 to severe^7,10^. WHO declared the end of the COVID-19 public health emergency of international concern on May 5, 2023, but the U.S. federal act on medical counter measures against the lingering threat of COVID-19 outbreaks remains in place until December 2024^11–13^. Therapeutics to treat COVID-19 thus continue to be important given the inequities in access to vaccines, the presence of variants, and the low to absent immune response to vaccines^14–17^. With the many clinical trials that have provided information about the effectiveness of treatments for COVID-19, recent research has shown that primary outcomes and other key information from the trials were not completely reported or had weak evidence about the therapeutics^18–21^. We would like to extend the results of prior evaluations in the context of an on-going need for COVID-19 therapeutics to include an assessment of the reporting of COVID-19 trial data in ClinicalTrials.gov and publications. The WHO and International Committee of Medical Journal Editors (ICMJE) require for completion of WHO Trial Registration Data Set (TRDS) items for basic trial information^22–24^. We aimed to assess completeness and changes to WHO TRDS data in trials on pharmacological interventions to treat COVID-19 registered on ClinicalTrials.gov and corresponding publications in peer-reviewed journals.

## Methods

We followed the Strengthening the Reporting of Observational Studies in Epidemiology (STROBE) reporting guidelines for the reporting of this cross-sectional study^25^ (Related File). The study was registered in the Open Science Framework platform (10.17605/OSF.IO/XH9AC).

### Study periods and data sources

This cross-sectional study on RCTs on COVID-19 pharmacological interventions comprised RCTs registered on ClinicalTrials.gov. Our study focuses on RCTs registered from January 1, 2020, and updated on May 31, 2021, along with any corresponding publications in peer-reviewed journals. This time frame captured the initial and critical phases of the COVID-19 pandemic, starting from the first reported case in December 2019 and extending through the early months of widespread clinical trials aimed at developing pharmacological and biological interventions. In ClinicalTrials.gov, we searched for the terms *COVID-19, SARS-CoV-2, coronavirus, pharmacological interventions,* and *biologicals* for interventional trials with results. Inclusion criteria for our sample were Food and Drug Administration Amendments Act (FDAAA) covered, which included interventional RCTs at all recruitment statuses with results posted, trials that involved pharmacological and biological interventions to treat COVID-19 disease and trials with corresponding publications in peer-reviewed journals included to make a comparison of registered data with published results. We searched PubMed, Web of Science, Google Scholar, and Scopus with the study title and identifier (NCT) for corresponding full-text publications in peer-reviewed journals regardless of whether they were in ICMJE member journals (Supplementary Material). Publication dates were determined using online publication dates for articles made available ahead of printing^26^. The investigation included trials initiated after the first reported case to the WHO with a search period that allowed at least one year for results submission and publication.

**Fig. 1:**
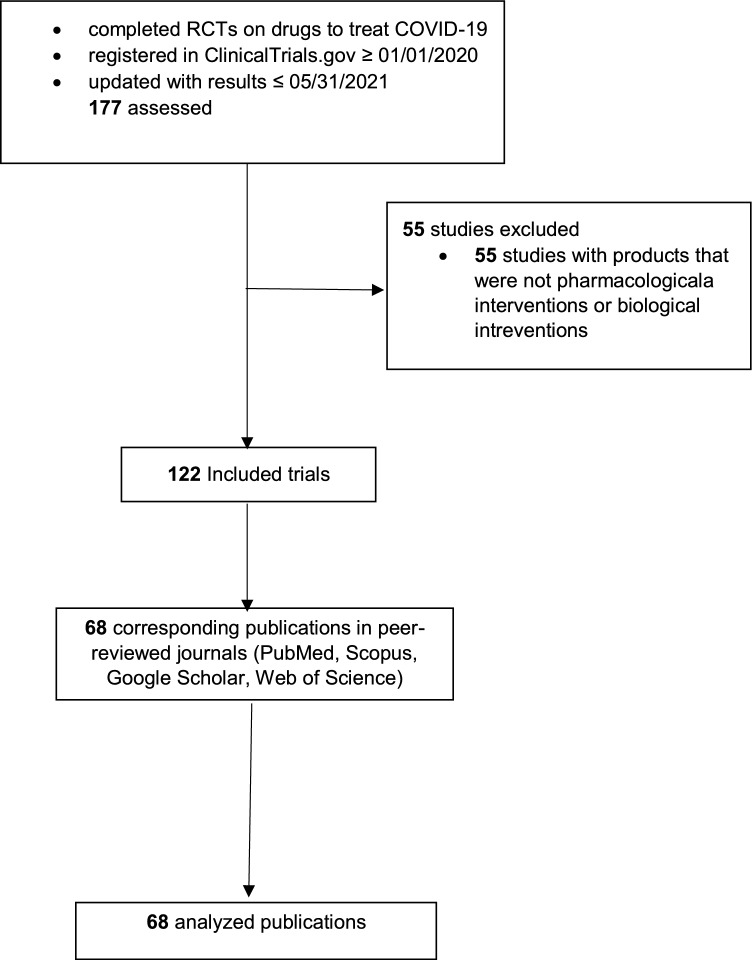
Study flow diagram.

We excluded trials that studied products that were not pharmacological interventions and biologicals, non-RCT studies (including observational studies or cohort studies), protocols and non-results trials, editorials and reviews, and preprints (Fig. 1).

### Data extraction and comparisons

We extracted trial data and WHO items from ClinicalTrials.gov and assessed their completeness at the initial and last registration. We additionally assessed changes from the initial to the last registration and between the latest registered data and publications based on previous methods^27^.

MS and SP independently abstracted data from the registry ClinicalTrials.gov and corresponding publications. This independent extraction aimed to minimize biases. Following the initial data extraction, the two authors held a consensus discussion to resolve any disagreements that occurred during their independent evaluations without the involvement of a third author. Consensus discussions were held to resolve disagreements concerning trial descriptions about the condition, funding source, and study design characteristics for which we achieved moderate agreement^28,29^. The completeness of WHO TRDS items was deemed as missing information from certain fields or uninformative terminology^27^. We used the criteria to assess discrepancies in the reporting of clinical trial data by Chan et al. Changes involved qualitative changes including differences in the meaning of the information provided in a registry field or quantitative changes such as differences in numerical entries^30^. The reliability of extractions concerning major changes for the two raters was high (kappa range: 0.80–1.00), where the lowest (kappa = 0.80, 95% CI 0.59–1.00) was for the changes in intervention descriptions between the latest registered data and data in publications. Data extracted from ClinicalTrials.gov was coded according to a calibrated coding key and then entered into a Microsoft Excel spreadsheet.

We used the categorizations of the WHO Data items on ClinicalTrials.gov to analyze differences in the frequency of uninformative items and changes in RCTs with at least 1 uninformative or changed item at the different time points according to categories: tracking information (registration and start dates), outcome measures, trial descriptive information (study design data), recruitment information (eligibility criteria, sample size, and study site information), and administrative information (identifying information, individual patient data availability, and funders). We did not seek approval from an institutional review board, as this study did not include participants or data that could identify individual participants.

### Statistical analysis

We assessed eligible trials for discrepancies in the completeness between the first and last registration of the WHO TRDS items and differences in the changes and completeness between the last registration and subsequent publications of the WHO TRDS items. Differences in the frequency of these discrepancies in the WHO TRDS items were compared using the Chi-square test. Post-hoc analyses were conducted using Bonferoni correction to adjust for multiple comparisons. Percentages, frequencies, or medians with 95% confidence intervals (CI) were presented. We considered a *P* < 0.05 to indicate statistical significance. We used IBM SPSS Statistics for Windows, version 21 (IBM Corp., Chicago, IL, USA) for the analysis.

## Results

### ClinicalTrials.gov trials characteristics

The initial extraction included 177 RCTs that met our inclusion criteria. We selected 122 eligible RCTs after applying exclusion criteria. We excluded 55 RCTs that studied products that were not pharmacological or biological interventions. Of those 122 RCTs, 68 had a corresponding publication in peer-reviewed journals (Fig. 1).

Most trials did not start before registration (n = 75/122, 61%). Most trials at initial registration were double-blind (n = 43/122, 35%), not industry-sponsored (n = 69/122, 57%), and were primarily for treatment (n = 101/122, 83%). Drugs were the most common intervention (n = 82/122, 67%) at initial and last registration, mostly in parallel assignment (n = 97/122, 80%) at initial registration, controlled by placebo (n = 62/122, 51%), and in phase 2 (n = 55/122, 45%) (Table 1.) Both male and female participants were in 118/122 (97%) RCTs.

**Table 1:** Study characteristics of COVID-19 RCTs in ClinicalTrials.gov in this study.

	First registration (n = 122)	Last registration (n = 122)
Phase, n (%)		
N/A	3 (2)	3 (2)
1	14 (12)	12 (10)
½	6 (5)	7 (6)
2	55 (45)	54 (44)
2/3	9 (7)	7 (6)
3	31 (26)	36 (30)
4	4 (3)	3 (2)
Masking		
Open label	39 (32)	39 (32)
Single blind	9 (7)	6 (5)
Double blind	43 (35)	44 (36)
Triple blind	8 (7)	11 (9)
Quadruple blind	23 (19)	22 (18)
Control		
Placebo	62 (51)	62 (51)
Active	33 (27)	31 (25)
Both	13 (11)	15 (13)
No control group	9 (7)	9 (7)
Not receiving anything	5 (4)	5 (4)
Assignment		
Single group	11 (9)	9 (7)
Parallel	97 (80)	101 (84)
Cross-over	3 (2)	2 (1)
Factorial	5 (4)	3 (2)
Sequined	6 (5)	7 (6)

RCTs—Randomized Controlled Trials.

### Completeness of WHO trial registration data set items

At the initial registration, the Secondary Identifying Number (UTN) item was missing for 95/122 (78%) RCTs (Table 2). None of the WHO items were found in another registry field at the initial registration (Table 2).

**Table 2:** Missing WHO TRDS Items in ClinicalTrials.gov concerning study identification, data sharing, and funder.

WHO item	Initial registration (n = 122)	Last registration, (n = 122)	Publication (n = 68)
Secondary identification number, n (%)	95 (78)^a^	1 (0.8)	39 (57)^b^
IPD Sharing statement, n (%)	23 (19)^c^	17 (14)^d^	52 (76)
Secondary sponsor, n (%)	0 (0.0)	0 (0.0)	6 (9)^e^
Sponsor/funder, n (%)	0 (0.0)	0 (0.0)	14 (21)^f^

WHO- World Health Organization; TRDS- Trial Registration Data Set; IPD- Individual Participant Data.

^a,b^*P* < 0.001 vs. last registration, Bonferroni post-hoc test. ^c,d^*P* < 0.001 vs. publication data, Bonferroni post-hoc test. ^e^*P* = 0.002 vs. initial and last registration, Bonferroni post-hoc test. ^f^*P* < 0.001 vs. initial and last registration, Bonferroni post-hoc test.

At the last registration, before publication, the Individual Patient Data (IPD) Sharing Statement was the predominantly (n = 17/122, 14%) missing item (Table 2).

As in the initial registration, none of the WHO items were found in another registry field at the last registration.

### Changes to WHO trial registration data set items during the trial from the initial to last registration

There were changes during the conduct of the trial to 19 of 24 WHO TRDS items for 122 RCTs. The most frequent major changes in RCTs from the initial to last registration were in the study completion date (97%), primary completion date (95%), and secondary identification number (90%). Recruitment and study description information items underwent more changes from the initial to the last registration than last registration to publications (Table 3).

**Table 3:** Changes in WHO TRDS items regarding study descriptions in ClinicalTrials.gov.

Changes in WHO TRDS items	Initial to last registration (n = 122)	Last registration to publication (n = 122)
Brief title	40 (33)	0 (0)
Official title	25 (20)	68 (56)
Study design	35 (29)	49 (40)
Condition	21 (17)	20 (16)
Intervention	76 (62)	2 (2)

WHO- World Health Organization; TRDS- Trial Registration Data Set; RCTs- Randomized Controlled Trials.

More changes were made to both primary and secondary outcomes from initial to last registration compared to changes made from last registration to publication [Chi-square value = 10.758, *P* = 0.001] (Table 4).

**Table 4:** Changes in key primary and secondary outcomes in ClinicalTrials.gov.

Changes in WHO TRDS items	Initial to last registration (n = 122)	Last registration to publication (n = 122)
Primary outcome, n (%)	87 (71)	9 (7)
Secondary outcome, n (%)	153 (125)	1 (1)

### Completeness of WHO trial registration data set in publications

Published articles were missing IPD sharing statements and secondary identifying numbers for 52/68 (76%) and 39/68 (57%) RCTs (Table 2).

### Changes to WHO trial registration data set items from the last registration to publication

The most dominant change from the last registration to publication was for Item 10 (Scientific Title) due to more information about study design in the registry than in the article for 68 publications (Table 3). Item 21 (Ethics Review) was present in 34/68 (50%) publications. Changes in Item 3 (Secondary Identifying Number) were recorded for 62/68 (91%) publications, mostly it was not listed in the article (39/68, 57%). Moreover, the dominant change involved registered primary outcomes omitted for 16/68 (24%) articles, and registered secondary outcomes omitted for 24/68 (35%) articles.

## Discussion

In the context of the ongoing need for therapeutics to treat COVID-19, we conducted a cross-sectional study to determine the completeness and changes to registration items disseminated in ClinicalTrials.gov and peer-reviewed publications. The frequency of discrepancies in essential WHO items for the proper reporting of patient data was high. Our study demonstrated the discrepancies in reporting for 122 RCTs conducted during the COVID-19 pandemic. The reason for inconsistent reporting may be the exponential rise of publications^31^ in journals^31–33^ which underwent pressure to expedite the review process^34^ four months after the WHO declared COVID-19 as a global health emergency^35^. Our study confirmed that the reporting of pharmacological interventions to treat COVID-19 was inconsistent as the quality of those publications in the early stages of the pandemic^32,33,36,37^. Research has consistently shown that industry-funded trials are more likely to exhibit selective reporting compared to non-industry trials. The finding from the current study has a similar trend. The lack of transparency in data sharing may be more shown in trials with commercial interests, potentially influencing the integrity of reported results. Our study indicates that selective reporting remains an issue in journals with high-impact factors^27,38^ which goes along with the findings of Zdravkovic et al.^39^ who revealed the quality of the COVID-19 publications in the three highest-ranked scientific journals below the quality average of these journals. Also, some authors confirmed the methodological quality of living systematic reviews of COVID-19 was reported low or critically low for the first time by Luo et al.^36^ and later by McDermott et al.^40^. Our study showed that the IPD-sharing statement was the predominantly missing item at the last registration (14%) and publications were missing IPD-sharing statements for 52 corresponding publications which confirmed that the IPD data-sharing statement remains accessible for a small number of RCTs conducted during the COVID-19^41,42^ even though it became an ethical obligation^43^ and requirement^44^ for clinical trials to be published in ICMJE journals. The expanded number of new publications during the pandemic required rapid ethics reviews^45^ but still, our study showed that 50% (n = 34/68) of publications reported about ethics committee approval. Additionally, we found more detailed and descriptive information in the registry than in the article regarding participant assignment, study phase, and the interventional model.

Our finding concerning the inconsistency in the reporting of essential data from COVID-19 trials corroborates with other studies^21^ that investigated completeness in reporting characteristics of nonsurgical periodontal therapy^46^ the completeness and changes concerning safety data from allergic rhinitis trials^47^ and the completeness of the reporting from drug-drug interaction trials^48^. There are several limitations to this study. First, the studies to be selected and used for data extraction from ClinicalTrials.gov may not be a comprehensive representation of the total set of COVID-19 trials in registries around the world. Moreover, recent findings showed that the majority of clinical trials concerning COVID-19 are registered in the Chinese Clinical Trials Registry^49^, so we may have excluded some trials. Second, due to the retrospective study design, data interpretation could be subjective, particularly for the qualitative data collection portion of the study where major changes in the WHO TRDS and results are determined. Additionally, we searched ClinicaTrials.gov and peer- reviewed journals in bibliographic database prior to registering the studyon OSF platform, so this can be considered as a limitation. However, the reliability of the extractions concerning major changes for the two raters was high (kappa range: 0.80–1.00), where the lowest (kappa = 0.80, 95% CI 0.59–1.00) was for the changes between the latest registered data and data in publications. Additionally, some investigators did not provide a registry number, so there was no possibility to find a corresponding publication. Disagreements in the independent extractions were not resolved by a third reviewer, so this might be a limitation to this study. Moreover, we excluded preprints from our study. Additionally, although we did not use the PRESS guideline for the peer review of our literature search strategies, which could be a limitation to this study, our searches used unique, study-specific information to find publications.

There is an urgent need to improve the reporting of COVID-19 trial data through concrete policy recommendations involving multiple research stakeholders. Training programs and educational interventions for researchers could be effective to improve the integrity of data sharing and publication ethics.

## Conclusions

This cross-sectional study established that inconsistent reporting of essential data in ClinicalTrials.gov and publications remained an issue despite professional guidelines. Discrepancies were relatively high for WHO TRDS items in ClinicalTrials.gov. We found that the majority of publications were missing IPD data sharing statements which contends with data sharing recommendations. There is also a need to highlight the discrepancies in key secondary outcomes followed by inclusion and exclusion criteria. Additionally, statements regarding ethics review were missing in the majority of publications. Incomplete reporting of patient and study design data from trials on pharmacological interventions to treat COVID-19 may compromise the reliability of data used in treatment decisions surrounding COVID-19 disease. There is a need to improve reporting consistency and transparency by educating researchers and improving policies.

## Supplementary Information

Supplementary Information.

## Data Availability

The datasets supporting the conclusions of this article are available in the Open Science Framework repository [https://osf.io/xh9ac/], 10.17605/OSF.IO/XH9AC.

## Abbreviations

COVID-19: Coronavirus disease 2019
EMA: European Medicines Agency
FDA: U.S. Food and Drug Administration
FDAAA: Food and Drug Administration Amendments Act
ICMJE: International Committee of Medical Journal Editors
IPD: Individual participant data
NCT: National Clinical Trial
RCT: Randomized controlled trial
SARS-CoV-2: Severe Acute Respiratory Syndrome Coronavirus 2
STROBE: Strenghtening the reporting of observational studies in epidemiology
TRDS: Trial Registration Data Set
UTN: Universal Trial Number
WHO: World Health Organization
