# Supplementary material for: Completeness and changes in data reporting pharmacological interventions to treat COVID-19
Source: Sci Rep. 2025 Jul 2;15:22989. doi: 10.1038/s41598-025-06308-y (PMC12215962; doi:10.1038/s41598-025-06308-y)
Supplement: Supplementary file 1 — Supplementary Information. [file 41598_2025_6308_MOESM1_ESM.pdf]

Search strategy for ClinicalTrials.gov (RCTs registered from January 1, 2020, and updated on May 31, 2021).

Search strategy in the Advanced Search feature:

terms: COVID-19, SARS-CoV-2, coronavirus, pharmacological interventions, biologicals

study phase: all study phases

study type: interventional type

study results: with results

Search strategy for publications in PubMed, Scopus, Google Scholar, and Web of Science using the study title and NCT identifier number from January 1, 2020, and updated on May 31, 2021

Search strategy for PubMed: ("Study title") OR (NCT number)

Search strategy for Scopus: ("Study title") AND (NCT number)

Search strategy for Google Scholar: ("Study title") AND (NCT number)

Search strategy for Web of Science: ("Study title") AND (NCT number)
